# Unveiling the invisible genomic dynamics

**DOI:** 10.1038/s12276-025-01434-z

**Published:** 2025-07-31

**Authors:** Jiwoong Kwon, Yeji Park, Taekjip Ha

**Affiliations:** 1 Program in Cellular and Molecular Medicine, Boston Children’s Hospital, Boston, MA USA; 2 Department of Pediatrics, Harvard Medical School, Boston, MA USA; 3 Howard Hughes Medical Institute, Boston, MA USA

**Keywords:** Fluorescence imaging, Chromatin structure

## Abstract

CRISPR-based imaging technologies have emerged as powerful tools for visualizing specific genomic loci, providing groundbreaking insights into chromatin structure and dynamics. Here, in this Review, we discuss the development and recent advances in these techniques, highlighting key strategies such as signal amplification, background reduction, multiplexing and enhanced genomic resolution. By engineering Cas proteins and guide RNAs, and incorporating peptide and aptamer tags, researchers have remarkably improved the sensitivity, specificity and resolution of CRISPR-based imaging, enabling the detection of nonrepetitive genomic regions and single-nucleotide polymorphisms. Recent studies have further pushed the boundaries of CRISPR-based imaging with the introduction of degron-mediated fluorogenic labeling and light-controllable background reduction. Despite remaining challenges, such as the bulkiness of signal amplification systems, limitations in guide RNA design and the effects of fixation on chromatin–protein interactions, CRISPR-based imaging holds great promise for advancing our understanding of chromatin dynamics, genomic interactions and their roles in various biological processes.

## CRISPR Imaging Transforms Understanding of Chromatin Dynamics

CRISPR technology, adapted from bacterial immune system, has ushered in a new era of genome editing and gene regulation through programmable genome targeting. Researchers have also developed CRISPR-based imaging technologies to better understand chromatin structures and dynamics in cells by visualizing specific DNA regions. This review covers the latest developments in these imaging techniques, focusing on methods to enhance signal strength, reduce background noise, and improve genomic resolution. Although there are still challenges, such as the complexity of the systems and limitations in guide RNA design, CRISPR-based imaging is a promising tool for understanding DNA dynamics and interactions in various biological processes.

This summary was initially drafted using artificial intelligence, then revised and fact-checked by the author.

## Introduction

Chromatin is a complex network of DNA interacting with various RNAs and proteins found in the eukaryotic nucleus^1–6^. It allows efficient compaction of genomic materials within the nucleus, maintaining genomic stability and regulating gene expression. The local structure of chromatin, which varies from the tightly packed heterochromatin to the more relaxed euchromatin, determines the accessibility of DNA to transcriptional machinery and other binding factors. Posttranslational histone modifications and chromatin remodeling complexes regulate the dynamics of chromatin, which plays an important role in DNA replication, damage repair and cell differentiation^7,8^. Although understanding these structural variations and their implications is vital for advancing gene regulation and developing therapeutic strategies, the lack of proper techniques has hindered the investigation of genomic dynamics in live cells.

The development of CRISPR (clustered regularly interspaced short palindromic repeats) technology, a groundbreaking advancement in genome editing, has opened new avenues for studying the dynamics of target genes. Over the past dozen years, the CRISPR system has revolutionized genetics by enabling precise, targeted modifications of DNA and efficient modulations of gene expression^9–11^. Originating from bacterial immune systems, this technology uses different endonucleases (CRISPR-associated proteins, Cas), with the specific endonuclease protein determined by the class and type of the CRISPR system, to cleave target nucleic acid sequences guided by a small RNA fragment (guide RNA, gRNA). The most widely used class 2 type II CRISPR–Cas9 system, which was derived from *Streptococcus pyogenes*, targets double-stranded DNA (dsDNA) in the genome to create dsDNA breaks, and resulting DNA damage response allows the insertion, deletion or replacement of genetic material with remarkable accuracy. Subsequent technical advancements, such as CRISPR interference, CRISPR activation and base and prime editors, have further enabled more precise genomic modifications and gene regulation with minimized side effects^12–16^. The versatility of the CRISPR–Cas9 system spans both basic research and therapeutic applications, providing a powerful tool for exploring gene function and its relationship to diseases.

Mutagenesis on Cas proteins has further initiated a new era for the visualization of specific genomic regions in real time. The development of nuclease-deactivated Cas9 (dCas9) protein enables the sequence-specific and stable binding of dCas9 in genomic regions, which can be readily visualized by fusing fluorescent proteins (FPs) to dCas9 (refs. ^16,17^). The combination of CRISPR–Cas9 and advanced labeling strategies allows the observation of changes in chromatin architecture and gene expression inside live and fixed cells with minimal perturbations^18^. These advances have greatly expanded the toolkit available for cellular imaging and biosensing, and facilitated the study of dynamic processes in gene regulation and chromatin remodeling. This Review will delve into these recent advancements, highlighting their impact on research and exploring future directions for further enhancing our understanding of gene function and regulation through CRISPR–Cas imaging technologies.

## Development of CRISPR-based imaging systems

The structure of DNA in situ has been routinely visualized by using DNA-staining organic fluorophores, FP-tagged DNA-binding proteins, DNA fluorescence in situ hybridization (FISH) and high-resolution imaging techniques such as atomic force microscopy and electron microscopy^19–24^. However, these conventional approaches have drawbacks when studying chromatin dynamics, such as disrupting cellular metabolism, lacking specificity and being incompatible with live specimens. More recently developed sequence-modulable systems, including zinc-finger proteins and transcription activator-like effectors, have provided the capability to image specific genomic regions of interest in live cells. However, they require complex protein engineering to alter the binding sequence, which is labor-intense, costly and time-consuming^25,26^. The CRISPR–Cas9-based imaging system was developed with the idea of overcoming the limitations of existing DNA-imaging technologies and providing easy access to any genomic regions of interest in live cells without disturbing their cellular metabolism.

CRISPR technology originates from the adaptive immune system of prokaryotes, where genetic information from invading pathogens is stored as short DNA sequences called spacers^27^. These spacers serve as a record for recognizing foreign genetic material upon future infections. The CRISPR array, composed of these spacers, is transcribed into pre-CRISPR RNA (pre-crRNA), which contains sequences complementary to the invading DNA, known as the protospacer. The pre-crRNA is then processed by either the Cas5 and Cas6 families (types I and III) or the housekeeping RNase III endonuclease (type II) into mature crRNA. Depending on the system, either a multiprotein complex (class 1) or a single effector protein (class 2) utilizes the spacer sequence in the crRNA to recognize and cleave the target gene^28,29^. A protospacer-adjacent motif (PAM) in the target DNA, typically consisting of three to eight base pairs located next to the protospacer sequence, plays a crucial role in the target recognition and cleavage by the CRISPR complex and serves as a key constraint in gRNA design. Type II systems require an additional *trans*-activating crRNA (tracrRNA) essential for the maturation of a functional gRNA, and the effector protein Cas9 facilitates and stabilizes the crRNA–tracrRNA duplex formation during the maturation, and proceeds to interfere with the target gene expression^30^. The inherent mechanism of CRISPR’s target recognition naturally allows flexible targeting of any genomic region in live cells, effectively overcoming the challenges associated with traditional DNA-imaging strategies.

**Fig. 1:**
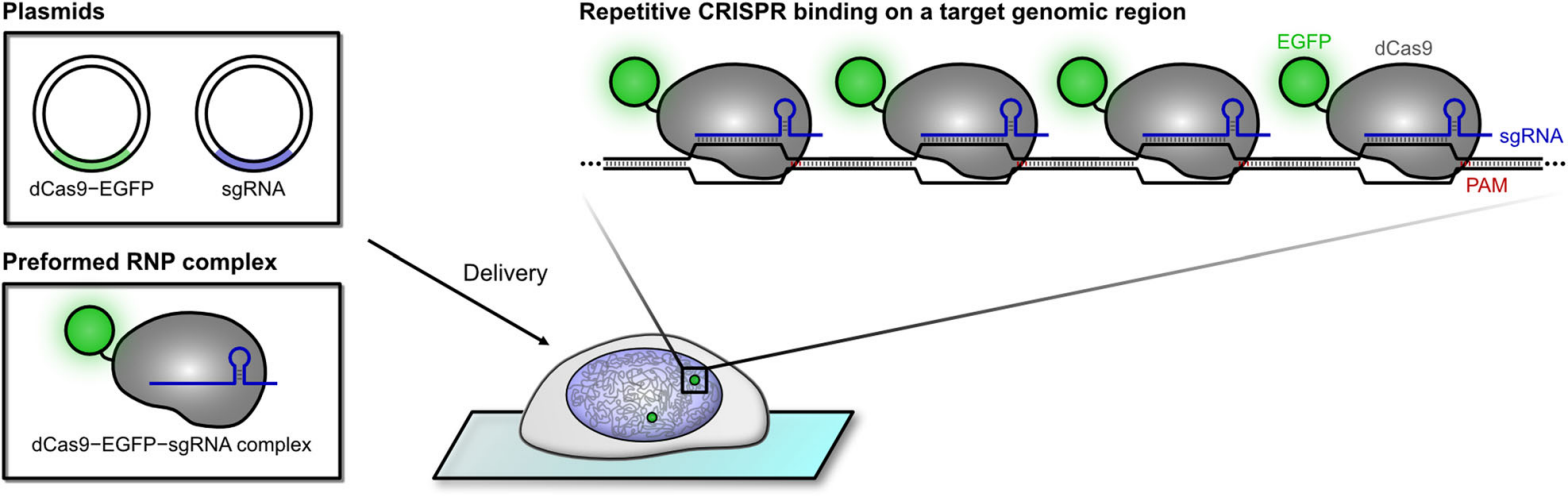
Schematic overview of the development of CRISPR–dCas9-based imaging system. The dCas9–EGFP fusion protein and sgRNA targeting specific genomic regions of interest can be delivered into the live cells as either plasmid DNA or preassembled RNP complex. Two point mutations in dCas9 preserve its DNA-binding ability while preventing cleavage. To simplify the system, a chimeric sgRNA is often used. Repetitive sequences are readily visualized due to the high local concentration of dCas9–EGFP, while nonrepetitive sites require signal accumulation strategies, such as tiling. Any genomic region can be imaged by altering the sgRNA spacer sequence, with only a minor restriction imposed by the PAM requirement, enabling cost-effective versatility.

The potential of CRISPR–Cas9-based imaging systems was first demonstrated in 2013, using FP-tagged dCas9 in combination with engineered chimeric single guide RNA (sgRNA), successfully visualizing the genomic loci in live cells (Fig. 1)^17^. The dCas9 mutant harbors two silencing mutations (D10A and H840A) in the nuclease domains (RuvC and HNH), which retain target specificity and tight binding capabilities but lack endonuclease activity^16^. To ensure efficient nuclear localization, two nuclear localization signal sequences were introduced at both the N- and C-termini of dCas9. Enhanced green fluorescent protein (EGFP) was added to the C-terminus of dCas9 after the nuclear localization signal sequence for visualization. A chimeric sgRNA, containing the protospacer, Cas9-binding hairpin and *Streptococcus pyogenes* transcription terminator, guided dCas9–EGFP to the target genomic loci^30^. To enhance the signal-to-noise (S/N) ratio, the sgRNA was engineered with an A–U base-pair flip to prevent premature termination by avoiding four consecutive U bases and extended hairpin structures to improve sgRNA–dCas9 assembly. Simultaneous delivery and expression of plasmids encoding dCas9–EGFP and sgRNA in various human cell lines enabled efficient visualization of genomic regions with repetitive sequences, such as telomeres and *MUC4*. By targeting multiple different sequences in a confined genomic region (~5 kb) near the target site, arbitrary nonrepetitive genes were also visualized with a sufficient S/N ratio, especially when ~30 sgRNAs were introduced. This method revealed the organization and dynamics of target loci in live cells without appreciably perturbing cellular activities or inducing DNA damage.

The CRISPR–dCas9-based imaging method has substantially advanced research on chromatin structure and dynamics, offering several advantages: sequence specificity, cross-species versatility, technical flexibility, ease of design, multiplexing capability and noninvasiveness. Over the past decade, subsequent technical developments have further enhanced this approach, leading to improved signal, higher sensitivity and simultaneous imaging of multiple genomic loci in live cells. These advancements have not only deepened our understanding of chromatin organization but also expanded the applications of CRISPR–dCas9 imaging in diverse areas of cellular and molecular biology.

## Signal amplification strategies

**Fig. 2:**
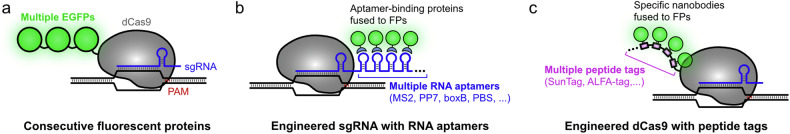
Signal amplification strategies. To enhance the S/N or S/B ratio, the CRISPR–dCas9 system amplifies fluorescence signals by recruiting multiple FPs to the target site. **a** In the simplest approach, a single dCas9 protein was engineered to carry up to three FPs. **b** Alternatively, sgRNA was modified to include multiple RNA aptamer sequences, which are recognized by their corresponding coat proteins fused to FPs. **c** Another strategy involved tethering multiple peptide tags to dCas9 instead of FPs. These tags were then labeled with specific nanobodies conjugated to FPs, enabling the recruitment of a higher number of FPs compared with direct FP tethering.

**Fig. 3:**
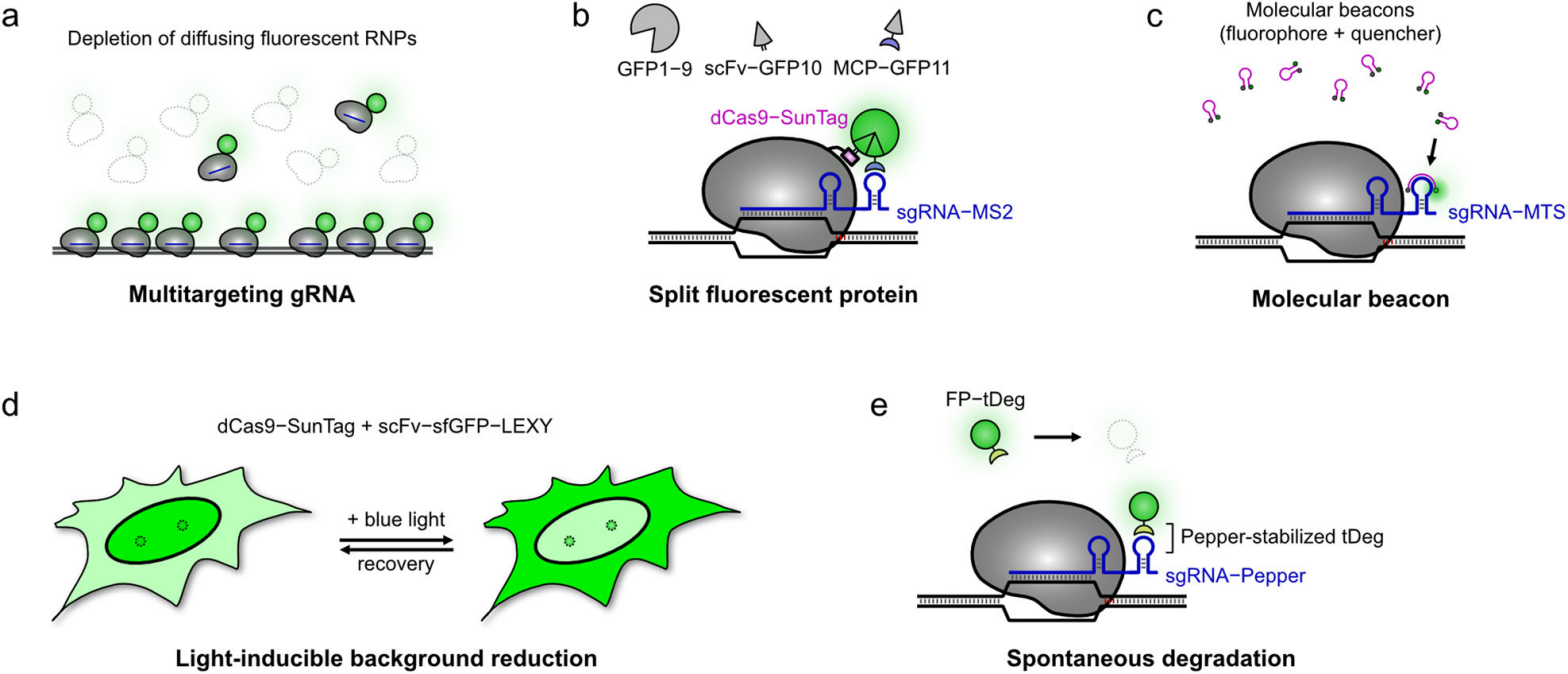
Strategies for reducing background signals to achieve higher contrast. As an alternative way to enhance the S/N or S/B ratio, the CRISPR–dCas9 system uses various approaches to reduce background fluorescence. **a** A multitargeting gRNA was designed to deplete background fluorescence from freely diffusing dCas9–EGFP molecules. This gRNA has over 300,000 potential binding sites across the genome, ensuring that most dCas9–EGFP molecules are stably bound to DNA rather than diffusing, enabling the detection of single dCas9–EGFP molecules. **b** A split GFP strategy was implemented using an engineered dCas9–SunTag and sgRNA–MS2 system. GFP fragments (GFP1–9, scFv–GFP10 and MCP–GFP11) assemble into a functional fluorescent GFP only when the RNP complex binds to the target DNA. Diffusing fragments remain nonfluorescent, effectively reducing background fluorescence. **c** A MB approach was introduced, utilizing an sgRNA engineered to incorporate a MTS. The MB, colabeled with a fluorophore and a quencher, remains quenched when unbound due to the close proximity of the fluorophore and quencher, effectively minimizing background signals. **d** A LEXY was combined with the dCas9–SunTag imaging system and scFv–sfGFP fusion proteins. Upon CRISPR complex binding to the target region, LEXY is activated during imaging to export unbound scFv–sfGFP molecules out of the nucleus, reducing background fluorescence. **e** A protein destabilizing motif (tDeg) was fused to the FP, which is stabilized only when bound to a specific RNA aptamer, Pepper. Unbound FP–tDeg molecules are rapidly degraded via the tDeg-mediated signaling pathway, leading to a drastic reduction in background fluorescence.

To efficiently detect genomic elements with low repeat numbers or visualize dynamic chromatin changes in live cells, imaging and labeling strategies have been developed to improve the S/N ratio in CRISPR–dCas9-based approaches. These strategies either amplify the signals from fluorescent probes or reduce background noise (Figs. 2 and 3). One straightforward method to enhance signal intensity involves tethering three consecutive copies of FPs direct to dCas9 (Fig. 2a)^31^. This approach has successfully visualized highly repetitive genomic regions, such as telomeres, pericentromeric and subtelomeric repeats, with a notable improvement in the S/N ratio. However, further increasing the number of FPs is challenging due to factors such as difficulties in constructing DNA plasmids, aggregation caused by misfolded FP, disruption to the protein degradation pathway and the induction of cellular stress^32–34^.

To further enhance fluorescence signals at target genomic regions, well-known RNA aptamers and their binding proteins labeled with FPs have been used through the facile modification of gRNA sequences (Fig. 2b)^35–37^. These hairpin-structured RNA aptamers have been commonly integrated into genome to study the transcription and translational dynamics^38–41^. By adapting this strategy, the CRISPRainbow system introduced three different RNA aptamers (MS2, PP7 and boxB), utilizing either two repeats of a single aptamer or a combination of two different aptamers within a single sgRNA^42^. By fusing three copies of FPs on the coat proteins, a higher S/N ratio was achieved, enabling the analysis on the dynamics of various genomic loci. Similarly, another group adopted the fluorogenic Broccoli aptamer, which becomes fluorescent upon binding to the small molecule DFHBI-1T, allowing the separate visualization of FP-labeled dCas9 and sgRNA to study their stability^43,44^. In another study using the 2×MS2 system, an optimized positioning of RNA aptamers substantially enhanced the S/N ratio, enabling the long-term tracking of genomic loci for over 1,000 s under continuous illumination^45^. Subsequent studies further increased the number of aptamers to up to 16 copies, using position optimization strategies, to provide real-time tracking of genomic loci dynamics for up to 4 h under optimized imaging conditions^46,47^. However, similar to FPs, an excessive number of aptamer copies can lead to lower sgRNA expression level and misfolded structures, reducing the binding efficiency of the coat proteins. To maximize sgRNA stability and coating efficiency, the CRISPR-Sirius system was designed with internal 8×MS2 or PP7 stem loops, where individual hairpin sequences were mutated to minimize transcript misfolding, demonstrating superior performance compared with the conventional 14×MS2 system inserted at the 3′ end^48^. CRISPR-Casilio utilized the conserved Pumilio/FBF (PUF) RNA-binding domain, which specifically binds to an 8-mer RNA sequence known as the PUF-binding site (PBS)^49^. This sequence is much shorter than other RNA aptamers (8 nt versus 20–30 nt). The PUF–PBS system demonstrated a well-correlated increase in signal intensity with the number of PBS repeats, up to 25 copies, leading to clear visualization of telomeres and centromeres.

As an alternative strategy, small peptide tags were introduced on the dCas9 protein, instead of RNA aptamers in the sgRNA, to enable multiple fluorescent tags to bind to a single dCas9 protein (Fig. 2c). One of the most well-known peptide tags is the SunTag, consisting of a 19-amino-acid GCN4 sequence that can be recognized by a specific single-chain variable fragment (scFv) antibody^32^. In the CRISPR-SunTag system, dCas9 was tagged with 24 repeats of the GCN4 sequence, recruiting multiple FPs to the dCas9 protein^50^. Each scFv antibody was labeled with either one or three copies of FPs, with the latter significantly enhancing signal intensity and improving the detection of telomeres and low-repetitive sequences. More recently, a 13-amino-acid peptide forming a stable α-helix, the ALFA-tag, was developed, inspired by an artificial peptide sequence^51^. The ALFA-tag is recognized by a specific nanobody (NbALFA), which carries fluorescent labels for imaging. A recent study attached up to 12 copies of ALFA-tags to the dCas9 protein, with split insertion of 6×ALFA at both the N- and C-termini providing higher signal intensity compared with having all 12 copies in the same terminus^52^. Expression of mRuby–NbALFA allowed direct visualization of ALFA-tagged dCas9 proteins targeting telomeres in living cells.

## Reducing background signals

In CRISPR–dCas9-based imaging systems, most dCas9–gRNA ribonucleoprotein (RNP) complexes or their binding partners are freely diffusing in the nucleus, with only a small fraction binding to target regions. Because these RNP complexes or their associated proteins are typically labeled with fluorescent tags, they contribute to background signals that obscure clear visualization of the true signal. A multitargeting gRNA was introduced to reduce background signals in the nucleus by directing most RNP complexes to the genome, leaving only a small fraction of fluorescent molecules diffusing in the nucleoplasm (Fig. 3a)^53^. The multitargeting gRNA binds to the B2 type short interspersed nuclear elements, which are repeated approximately 350,000 times throughout the mouse genome^54^. The dCas9 protein was fused to a HaloTag domain, which can be recognized by a cell-permeable HaloTag ligand labeled with organic fluorophores^55,56^. With a short exposure time (10 ms), individual dCas9 proteins were visualized owing to the low background levels, enabling the investigation of dCas9’s motional dynamics at target sites and off-target binding events.

Other groups have utilized an optimized split protein tag to reduce the background signal. Split GFP, particularly the 11th β-strand (GFP11) and the rest (GFP1–10) of superfolder GFP (sfGFP), offers a cost-efficient and scalable method for tethering to specific proteins due to the small size of GFP11 (refs. ^57,58^). In the CRISPR-Tag system, 14 copies of GFP11 were directly inserted in the dCas9 protein, and the co-expressed GFP1–10 enabled visualization of dCas9 (ref. ^59^). This approach yielded a threefold increase in S/N ratio compared with dCas9–EGFP, although the background level was not sufficiently reduced, probably because freely diffusing dCas9–(GFP11)_14_ complexes could still form fully assembled sfGFP molecules. To improve this approach, the tripartite split GFP system was combined with RNA and peptide tags (Fig. 3b)^60,61^. The dCas9 protein was tagged with 24 repeats of the SunTag peptide, while the sgRNA was modified to include 12 copies of MS2 hairpins. The binding partners, scFv antibody and MCP protein, were labeled with GFP10 and GFP11, respectively, while the remaining GFP1–9 was co-expressed within the cell. As a result, sfGFP assembly became highly efficient only when the RNP complex bound to the target genomic region, forming a stable structure, which led to an over tenfold reduction in nuclear background signal.

Another group introduced a molecular beacon (MB), a small single-stranded (ss)DNA fragment labeled with a fluorophore and a quencher at its two termini^62^. In the CRISPR-MB system, the sgRNA was engineered to include a MB target sequence (MTS) at the second stem–loop structure; thus, the MB becomes fluorescent only when it binds on MTS (Fig. 3c)^63^. This system demonstrated superior sensitivity in detecting telomere spots and better photostability compared with EGFP-tagged telomere-binding protein TRF1, without disrupting the motional dynamics of the telomeres. This approach was further refined by using two different MB fragments, known as CRISPR-dualFRET (fluorescence resonance energy transfer)^64^. In this system, a FRET donor was attached to one MB and an acceptor to the other MB, and the MTS was modified to allow binding of both MBs, inducing FRET only when both MBs properly bound to the MTS. CRISPR-dualFRET improved the S/N ratio by approximately 3.5 times compared with CRISPR-MB, enabling the visualization of nonrepetitive loci using only three unique gRNAs.

Recent studies have introduced optogenetic control or spontaneous, rapid degradation of unbound fluorescent tags to reduce background level^65–67^. The CRISPR-based light-inducible background reduction (CRISPR-LIBR) system used a light-inducible nuclear export tag (LEXY) tethered to FPs (Fig. 3d)^65^. Upon blue light illumination, unbound FPs were quickly exported from the nucleus, resulting in an approximately sixfold increase in the S/N ratio. For targeted degradation of the unbound FPs, a bifunctional peptide (tDeg) was introduced on FPs (Fig. 3e)^68^. The tDeg peptide contains a degron sequence (RRRG), which is protected only when bound to the small RNA aptamer, Pepper. By engineering the sgRNA to include Pepper sequences in its structure, the S/N ratio improved by approximately 20-fold compared with the dCas9–EGFP system and 4-fold compared with the sgRNA–MS2/dCas9–MCP system^66,67^.

## Multiplexing

**Fig. 4:**
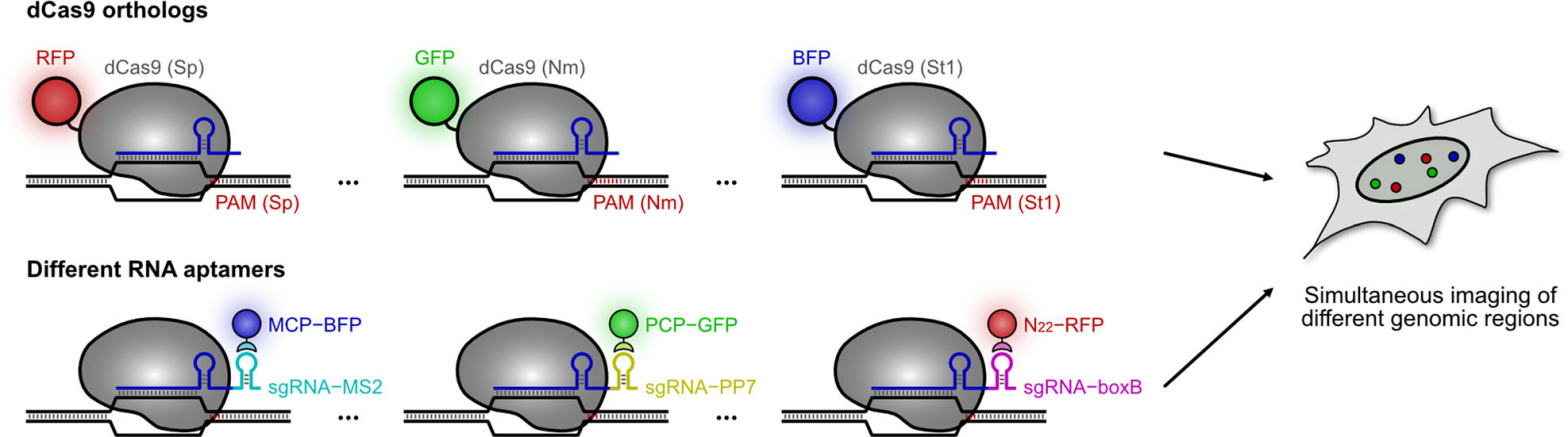
Multiplexing in the CRISPR–dCas9 system. To simultaneously visualize different genomic regions, multicolor approaches have been applied to the CRISPR–dCas9 system by utilizing dCas9 orthologs from different species or by combining RNA aptamers with their corresponding coat proteins. Top: dCas9 proteins derived from Sp, Nm and St1 were labeled with RFP, GFP and BFP, respectively. By designing sgRNAs to target different genomic regions with distinct PAM requirements, three separate genomic sites were imaged simultaneously. Bottom: alternatively, distinct RNA aptamers (for example, MS2, PP7 and boxB) were incorporated into sgRNAs targeting different genomic regions. These aptamers were paired with their respective coat proteins fused to FPs with separated spectral properties (for example, MCP–BFP, PCP–GFP and N22–RFP), visualizing multiple genomic regions at the same time.

The multiplexing capability of an imaging technique allows the interrogation of multiple structural details and their interactions. In CRISPR-based imaging methods, simultaneous visualization of two or more genomic regions has been achieved using different PAM requirements of dCas9 proteins from various species, or through labeling strategies designed to enhance the S/N ratio (Fig. 4). Ma et al. introduced three types of dCas9 protein derived from *Streptococcus pyogenes* (Sp), *Neisseria meningitidis* (Nm) and *Streptococcus thermophilus* (St1), each recognizing distinct PAM sequences (NGG, NNNNGATT and NNAGAAW for Sp, Nm and St1, respectively)^31,69^. By tagging each dCas9 protein with FPs of different spectral properties, they were able to simultaneously observe different genomic regions with high repeat numbers. This approach was further enhanced by combining it with the CRISPR-LIBR system to image chromosomes 3 and 7 together^65^. Similarly, RNA aptamers have been used to label multiple genomic regions, with their binding proteins fused to spectrally distinct FPs^42,45,46,48,49^. The CRISPR-MB system also expanded its imaging targets by introducing different MTS sequences and complementary MBs labeled with different fluorophores^63^. More recently, two fluorogenic methods, CRISPR-Broccoli and tDeg–Pepper-based targeted degradation, were combined to demonstrate multiplexing capability with a superior S/N ratio for low-repeat sequences on chromosomes 3 and 13 (ref. ^66^).

## Visualizing nonrepetitive genomic regions

**Fig. 5:**
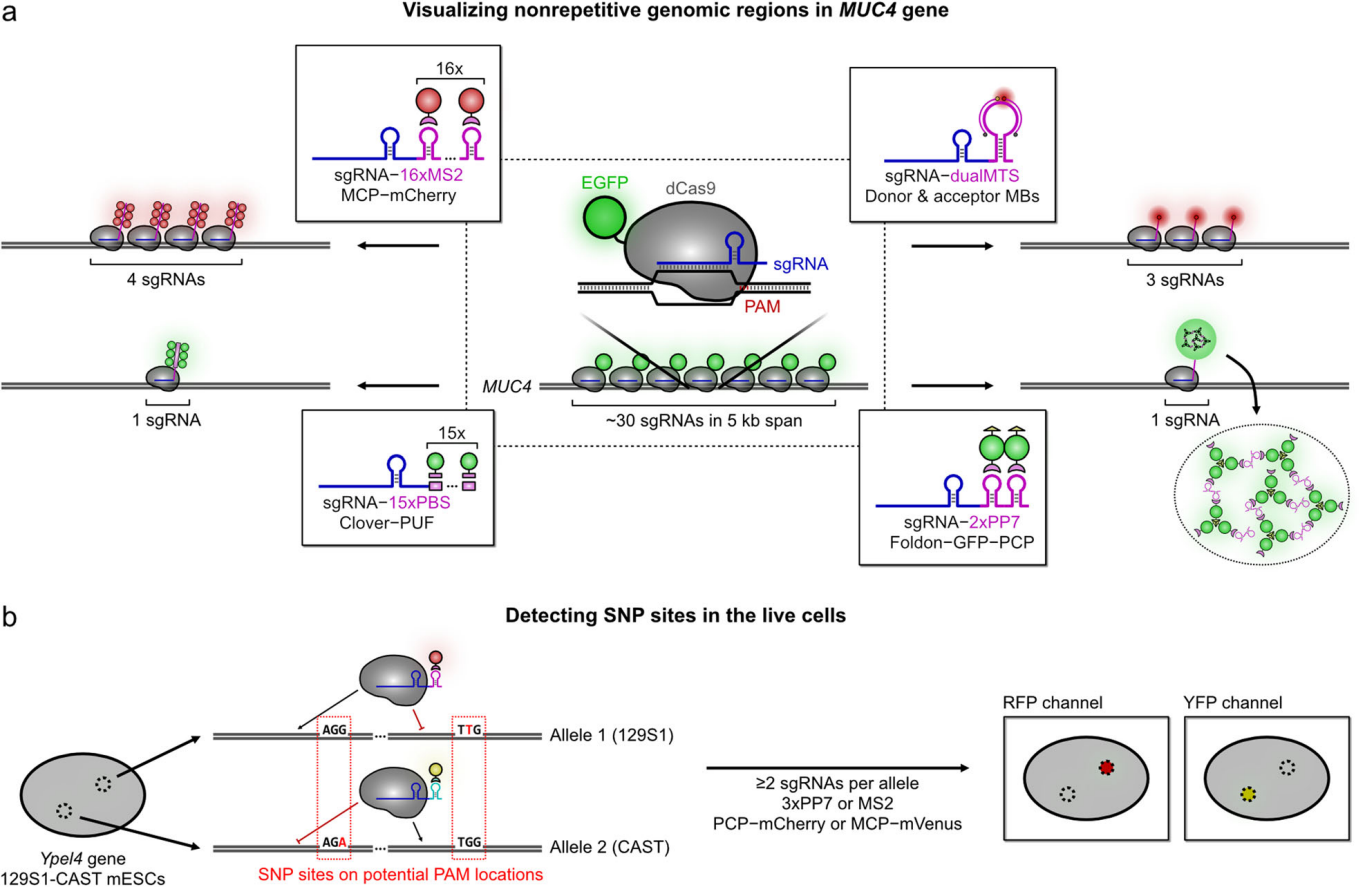
Strategies for enhanced genomic resolution. CRISPR–dCas9-based imaging techniques have been utilized to improve genomic resolution, enabling the visualization of nonrepetitive genomic regions and SNP sites. **a** Efficient visualization of nonrepetitive sequences. Early approaches demonstrated the feasibility of visualizing nonrepetitive sequences by introducing multiple sgRNAs (~30 sgRNAs) to a confined genomic region. Signal amplification strategies were subsequently adopted to reduce the number of sgRNAs required. The use of multiple MS2 stem loops in sgRNAs or MB-based FRET approaches enabled visualization of the same genomic region with only four or three sgRNAs, respectively. Introducing PUF or foldon allowed visualization of target sites with just a single sgRNA. **b** SNP site detection in living cells. The PAM sequence plays a critical role in target specificity, with point mutations in the second or third PAM positions effectively preventing RNP complex binding to the target gene. By designing sgRNAs to position SNP sites within PAM regions, allele-specific SNP mutations can be visualized in living cells.

One of the ultimate goals of CRISPR-based imaging techniques is the visualization of non-repetitive genomic loci, enabling the imaging of any genomic region of interest (Fig. 5a). This was first demonstrated by expressing multiple engineered sgRNAs targeting the intronic region of the *MUC4* gene, with binding regions confined to a ~5 kb span^17^. In this initial version of CRISPR-based imaging, the dCas9 protein was labeled with a single GFP molecule, requiring around 30 sgRNAs to successfully visualize the nonrepetitive target site. By utilizing 16 copies of RNA aptamers at optimized positions within the sgRNAs, highly amplified signals allowed detection of the same genomic loci (*MUC4*) with only four sgRNAs, using an advanced lattice light-sheet microscope that provided lower background levels compared with conventional widefield imaging techniques^47,70^. Further advancement came with the CRISPR-dualFRET system, which used a quencher and FRET-based strategy to effectively suppress background noise, reducing the required number of sgRNAs to just three^64^. This system successfully visualized nonrepetitive genomic regions of *MUC4*, *MUC1* and an intergenic region using only three unique sgRNAs per target site.

Recent advancements in labeling strategies have enabled the visualization of nonrepetitive loci using just one sgRNA^71,72^. Chow et al. improved the CRISPR-Casilio technique by expanding the number of PBS up to 25, allowing the recruitment of more FPs at target loci^49,71^. This method successfully visualized various genes, including *MUC4*, *MASP1* and *BCL6*, using a single sgRNA per gene. It revealed insights into chromatin structural dynamics, RAD21-dependent structural changes and loop-forming interactions between the promoter, gene body and superenhancer. Lyu et al. introduced a self-assembly-based signal amplification strategy called CRISPR-FISHer (FISH amplifier)^72^. This approach utilized the trimeric motif of T4 bacteriophage fibritin, foldon, which forms a stable trimer in solution^73^. The foldon was tethered on GFP–PCP fusion protein to allow the spontaneous trimer formation of foldon–GFP–PCP. With co-expressed sgRNA–2×PP7, the foldon–GFP–PCP trimers recruit freely diffusing sgRNA–2×PP7 through PCP–PP7 interactions, resulting in large cluster formations of foldon–GFP–PCP and sgRNA–2×PP7. For repetitive regions, CRISPR-FISHer provided a more than 20-fold enhancement in the signal-to-background (S/B) ratio compared with previous dCas9–EGFP and PCP–GFP techniques. This method was used to image the *ppp1r2*, *TOP1* and *TOP3* genes with one sgRNA per target, enabling the study of spatial relationships between genomic regions. In addition, they combined CRISPR-FISHer with CRISPR-Sirius to visualize a repetitive region on chromosome 3 alongside the *ppp1r2* gene, demonstrating intrachromosomal dissociation and rejoining mediated by the DNA damage response.

## Imaging at the single-nucleotide genomic resolution

The sequence specificity of CRISPR-based imaging methods allows for the visualization of virtually any genomic location, constrained only by the minor requirement of a PAM sequence^74–78^. To fully exploit this potential, Maass et al. developed an imaging strategy called SNP-CLING, which enables single-nucleotide genomic resolution for the detection of single-nucleotide polymorphisms (SNPs; Fig. 5b)^79^. They designed sgRNAs with protospacer sequences to have a SNP mutation at the second or third position of the PAM, allowing either the intact or mutated allele to bind to dCas9. The sgRNAs were also engineered with three RNA aptamer sequences to recruit coat proteins tagged with two FPs. Using three precisely designed sgRNAs per target gene, they achieved clear discrimination between alleles involving the SNP sites, allowing the study of interallelic distances, as well as their spatial relationships with nucleoli and the nuclear periphery over time. However, this approach still requires at least two sgRNAs per allele for a sufficient S/N ratio and, as a result, cannot be used to detect a SNP. Further efforts have focused on achieving single-nucleotide genomic resolution using a single sgRNA for allele-specific applications in fixed cells.

## CRISPR-based methods for fixed-cell imaging

**Fig. 6:**
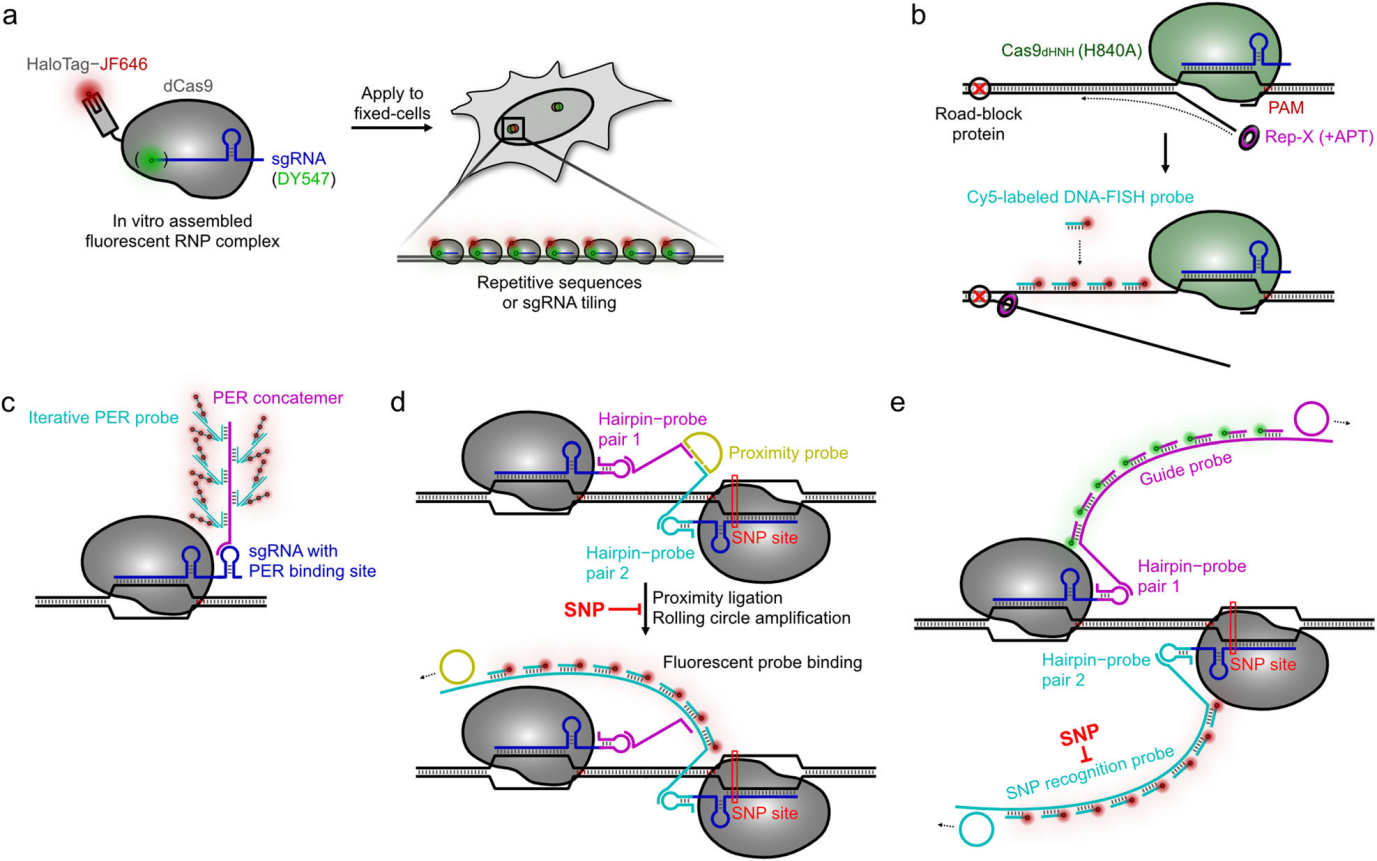
CRISPR–dCas9 technologies for fixed-cell imaging. **a** The binding of the RNP complex to target sites does not require a denaturation step for dsDNA. By applying a preassembled fluorescent RNP complex to fixed specimens, both repetitive and nonrepetitive genomic regions can be readily visualized using single or multiple sgRNAs, respectively, without disrupting chromatin organization. **b** The introduction of a Cas9 nickase (Cas9_dHNH_) and a superhelicase (Rep-X) enables local denaturation of dsDNA, facilitating the binding of conventional DNA-FISH probes. This approach allows any genomic region to be visualized with higher labeling density, free from the constraints of PAM sequences. **c** Signal amplification via an exchange reaction has been used to image nonrepetitive sequences using a single sgRNA. An engineered sgRNA containing a PER-binding site can recruit a PER concatemer, which can subsequently be labeled with PER probes in an iterative manner, enhancing signal intensity drastically. **d**, **e** SNP sites in fixed cells were efficiently detected using RCA. Two RNP complexes were designed to target genomically proximal regions, with one region potentially harboring an SNP mutation at the third position from the PAM. Each sgRNA was engineered to include a distinct hairpin structure, allowing recognition by corresponding probes. **d** A proximity probe was introduced, designed to bind the target region only when the two RNP complexes are correctly positioned. RCA elongates the probe tethered to the RNP complex at the potential SNP site, enabling visualization through fluorescent probe binding. SNP mutations disrupt the binding of one RNP complex, preventing RCA and eliminating the fluorescent signal. **e** Alternatively, distinct hairpin probes can initiate separate RCA reactions, removing the proximity restriction for the two RNP complexes. In this setup, SNP mutations result in the loss of one RCA reaction, enabling analysis by measuring the loss of colocalization between the signals.

Although one of the major advantages of CRISPR-based techniques is their compatibility with live-cell imaging, they also offer a way to overcome limitations of conventional DNA imaging methods used in fixed cells, such as DNA-FISH^20,80^. DNA-FISH requires a global denaturation step of the dsDNA, often involving heating the specimen to over 80 °C for extended periods, which can compromise the structural integrity and three-dimensional organization of the genome^81,82^. In addition, DNA-FISH relies on multiple fluorescent probes for each target region to achieve adequate detection, but using more probes can reduce genomic resolution. Owing to the stable binding properties of the RNP complex to its target dsDNA, the Cas9-mediated FISH (CASFISH) method was introduced as an alternative to preserve genomic structural integrity by eliminating the heat-induced global denaturation step required in DNA-FISH (Fig. 6a)^83^. CASFISH directly applied preassembled RNP complexes, consisting of a HaloTag-labeled dCas9 protein and sgRNA, to fixed specimens and successfully imaged various genomic regions, including low-repetitive sequences (intron 3 of *MUC4*) and nonrepetitive sites (first intron of *MUC4*) using 73 sgRNAs. This direct RNP-delivery labeling strategy was later adapted for live-cell imaging through the use of fluorescently labeled gRNA, known as CRISPR-LiveFISH^84^. Thanks to the increased brightness of organic fluorophores, CRISPR-LiveFISH achieved a fourfold enhancement in S/B ratio compared with dCas9–EGFP, and further enabled the study of DNA damage-induced cellular responses, such as the recruitment of repair proteins and chromosomal translocation.

Recent advances for CRISPR-based fixed-cell imaging have aimed to enhance genomic resolution, including the visualization of nonrepetitive sites and achieving single-nucleotide resolution. Wang et al. introduced an alternative DNA-FISH method that preserves genomic structure integrity by eliminating the global denaturation of dsDNA (Fig. 6b)^85^. They used engineered proteins, specifically Cas9 nickase (Cas9_dHNH_) and a superhelicase (Rep-X), to generate ssDNA breaks and achieve local denaturation via helicase activity^86,87^. Cas9_dHNH_, which carries a point mutation (H840A) in its nuclease domain, nicks only the nontarget strand, and Rep-X, an internally cross-linked version of Rep helicase, can unwind thousands of base pairs with high processivity. The combination of these two engineered proteins creates nicks at the target site, guided by gRNA, and these are locally denatured by the superhelicase to allow binding of fluorescent oligo probes. This method, termed genome oligopaint via local denaturation FISH (GOLDFISH), efficiently visualized nonrepetitive DNA sequences through tiled probe binding. It was further refined to achieve single-nucleotide sensitivity by introducing a specificity-enhanced RNP complex composed of an enhanced Cas9 nickase and a sgRNA with a 5′-extended guanine base^88–90^. With an intentional mismatch between the protospacer and target site, the high-specificity RNP complex was able to generate a nick on the target, while an additional mismatch strongly inhibited nickase activity, enabling the detection SNP sites in Hutchinson–Gilford progeria syndrome cells. To further boost signals from nonrepetitive sequences targeted by a single sgRNA, Li et al. developed a method called CasSABER (CRISPR–Cas9-mediated signal amplification by exchange reaction; Fig. 6c)^91^. This technique uses a presynthesized long concatemer, generated via a primer-exchange reaction (PER), which binds to an engineered sgRNA^92^. The PER probe recruits multiple fluorescent imager strands, resulting in substantial signal amplification. An iterative version of the PER probe allows multiple rounds of fluorescent imager hybridization, enabling the visualization of nonrepetitive sequences of the *MUC4* gene using just a single sgRNA.

For efficient SNP site detection, in situ rolling circle amplification (RCA) has been utilized in two imaging techniques: CasPLA (CRISPR–Cas9-mediated proximity ligation assay) and CoDeC (colocalization of dual-engineered CRISPR probes)^93,94^. Both methods use two gRNAs in proximity to recognize reference and SNP sites, with the SNP-mediated mismatch positioned in the PAM-proximal region. Each gRNA is engineered with an additional hairpin structure that serves as a binding template for intermediate DNA probes. In CasPLA, the proximity probe binds to the two intermediate DNA probes when they are spatially close, followed by proximity ligation to form a circular DNA structure for RCA (Fig. 6d). The resulting long RCA product is then labeled with multiple fluorescent probes for detection. RCA proceeds efficiently only when there is no mismatch on the gRNA targeting the SNP site, with a single nucleotide mutation causing the loss of the fluorescent signal. In CoDeC, each intermediate DNA probe is processed orthogonally for recognition by a padlock probe, followed by an RCA reaction (Fig. 6e). Each long RCA product is labeled with spectrally distinct fluorescent probes, enabling SNP variation evaluation through colocalization analysis. Both CasPLA and CoDeC have demonstrated their ability to image nonrepetitive genomic regions with high specificity for SNP mutations, including in the *KRAS* gene and mitochondrial DNA.

## Concluding remarks

In this Review, we have discussed the evolution and recent advances in CRISPR-based imaging technologies, emphasizing the technical strategies used to achieve specific goals, such as signal amplification, background noise reduction and improved genomic resolution. Techniques such as multiplexing, detection of nonrepetitive genomic regions and single-nucleotide resolution have been realized through the introduction of engineered Cas9 proteins and gRNAs, along with peptide and aptamer tagging, leveraging the sequence and species specificity of the CRISPR system. The CRISPR–dCas9-based imaging approach has demonstrated its potential in studying genomic loci dynamics^17,44^, interallelic interactions^79^, SNP detection^93^, DNA damage response^84,95^, chromatin compaction^96^ and chromatin loop structures^71^. Moreover, this technology has been expanded to detect other nucleic acid molecules, such as ssDNA and ssRNA, by incorporating additional CRISPR systems involving Cas13a and Cas14 proteins^97,98^.

Despite notable progress, several limitations have constrained the broader application of CRISPR-based imaging techniques in studying chromatin structure and related biological processes. For instance, the bulkiness of signal amplification systems may interfere with chromatin dynamics and the interactions of DNA-binding factors. In addition, the effectiveness of this approach heavily depends on the precise design of gRNAs and the efficient delivery of CRISPR components. Achieving a better S/N ratio and multiplexing capabilities often introduces increased complexity and reduced efficiency. Fixed-cell imaging methods rely on methanol- and acetic acid-based fixation for efficient probe binding, which alters protein structures thereby hindering the simultaneous study of chromatin and its interacting proteins. Although improved labeling strategies are needed to overcome these challenges for both live and fixed cells, CRISPR-based imaging has nonetheless opened up powerful opportunities to visualize and analyze specific genomic regions in live cells. When combined with advanced microscopy techniques, such as superresolution imaging, we anticipate that CRISPR-based imaging will soon enable the direct visualization of fine chromatin structure details, its dynamics and its interactions with other proteins.
